# Radiation-induced inflammation and autoimmune diseases

**DOI:** 10.1186/s40779-018-0156-7

**Published:** 2018-03-20

**Authors:** Rasoul Yahyapour, Peyman Amini, Saeed Rezapour, Mohsen Cheki, Abolhasan Rezaeyan, Bagher Farhood, Dheyauldeen Shabeeb, Ahmed Eleojo Musa, Hengameh Fallah, Masoud Najafi

**Affiliations:** 1 School of Medicine, Jiroft University of Medical Sciences, Jiroft, Zip code: 8813833435 Iran; 2 Department of Radiology, Faculty of Paramedical, Tehran University of Medical Sciences, Tehran, Zip code: 1417613151 Iran; 3 Department of Radiologic Technology, Faculty of Paramedicine, Ahvaz Jundishapur University of Medical Sciences, Ahvaz, Zip code: 6135715794 Iran; 4 Department of Medical Physics, School of Medicine, Iran University of Medical Sciences, Tehran, Zip code: 1449614535 Iran; 5 Departments of Medical Physics and Radiology, Faculty of Paramedical Sciences, Kashan University of Medical Sciences, Kashan, Zip code: 3715835155 Iran; 6 Department of Medical Physics and Biomedical Engineering, Faculty of Medicine, Tehran University of Medical Sciences (International Campus), Tehran, Zip code: 1417613151 Iran; 7 Department of Physiology, College of Medicine, University of Misan, Misan, Iraq; 8 Research center for molecular and cellular imaging, Tehran University of Medical Sciences, Tehran, Zip code: 1417613151 Iran; 9 Department of Chemistry, Faculty of Science, Islamic Azad University, Arak, Zip code: 3836119131 Iran; 10 Radiology and Nuclear Medicine Department, School of Paramedical Sciences, Kermanshah University of Medical Science, Kermanshah, Zip code: 6714869914 Iran

**Keywords:** Radiation, Inflammation, Autoimmune diseases, Thyroid, Diabetes

## Abstract

Currently, ionizing radiation (IR) plays a key role in the agricultural and medical industry, while accidental exposure resulting from leakage of radioactive sources or radiological terrorism is a serious concern. Exposure to IR has various detrimental effects on normal tissues. Although an increased risk of carcinogenesis is the best-known long-term consequence of IR, evidence has shown that other diseases, particularly diseases related to inflammation, are common disorders among irradiated people. Autoimmune disorders are among the various types of immune diseases that have been investigated among exposed people. Thyroid diseases and diabetes are two autoimmune diseases potentially induced by IR. However, the precise mechanisms of IR-induced thyroid diseases and diabetes remain to be elucidated, and several studies have shown that chronic increased levels of inflammatory cytokines after exposure play a pivotal role. Thus, cytokines, including interleukin-1(IL-1), tumor necrosis factor (TNF-α) and interferon gamma (IFN-γ), play a key role in chronic oxidative damage following exposure to IR. Additionally, these cytokines change the secretion of insulin and thyroid-stimulating hormone(TSH). It is likely that the management of inflammation and oxidative damage is one of the best strategies for the amelioration of these diseases after a radiological or nuclear disaster. In the present study, we reviewed the evidence of radiation-induced diabetes and thyroid diseases, as well as the potential roles of inflammatory responses. In addition, we proposed that the mitigation of inflammatory and oxidative damage markers after exposure to IR may reduce the incidence of these diseases among individuals exposed to radiation.

## Background

Every year, millions of people are exposed to ionizing radiation resulting from diagnostic or interventional radiology and radiotherapy. In addition, nuclear and radiologic disasters pose a threat to all individuals worldwide. In addition to the killing effects of ionizing radiation at high doses, exposure to sub-lethal doses may result in various diseases, such as carcinogenesis, cataract, and cardiovascular disease [1, 2].

Although genomic instability and carcinogenesis are the most important concerns of ionizing radiation, studies have revealed that exposure to IR can strongly affect immune system responses, leading to changes in the normal functions of immune responses [3]. These effects may be responsible for various diseases among exposed people. Studies have proposed that chronic inflammation and continuous free radical production are responsible for several diseases after radiotherapy or radiation accident. Several studies have proposed that 25% to 50% of all cancers may be related to chronic inflammation [4–6]. Moreover, continuous free radical production, resulting from inflammatory responses, can disrupt organ function. For example, chronic oxidative damage in the kidneys of individuals with diabetes is mediated by increased insulin-like growth factor 1(IGF-1) and nicotinamide adenine dinucleotide phosphate(NADPH) oxidase enzymes [7, 8]. This situation has been confirmed for other organs, such as Crohn’s disease and ulcerative colitis in the gastrointestinal system, pancreatitis, and rheumatoid arthritis [9–13].

Under normal conditions, there is a balance between the levels of free radicals and the antioxidant defenses that help prevent reactive intermediates from damaging cells and tissues. Free radicals play an important role in cell signaling; however, excessive amounts of reactive oxygen species(ROS), as observed following exposure to IR, can cause damage to cellular genetic contents, proteins, and lipids [14]. Free radicals have different types in cells, including superoxide (O2•-), nitric oxide (NO), and the hydroxyl radical (OH•). However, other types of molecular species, such as hydrogen peroxide (H_2_O_2_) and peroxynitrite (ONOO-), can be produced by IR interactions or subsequent metabolites [15, 16].

There is a strong relationship between chronic inflammation and oxidative damage after exposure to IR [17]. IR can alter the numbers and functions of immune system cells in irradiated organs. Increased numbers of macrophages and lymphocytes T (T-cells) induce the secretion of several inflammatory mediators, such as NF-κB and SMAD2/3, and cytokines, such as IL-1, IL-2, IL-6, IL-8, IL-33, tumor necrosis factor (TNF-α), transforming growth factor beta (TGF-β), and interferon gamma (IFN-γ) [18]. The elevated level of these inflammatory mediators is associated with the release of prostaglandins and free radicals, including reactive oxygen species (ROS) and nitric oxide (NO) [19]. Under conditions, such as exposure to high doses of IR during a radiation disaster, these inflammatory responses may continue for years after exposure [20]. In this situation, chronic inflammation and its consequences may disrupt the functions of irradiated organs [21].

## Radiation toxicity in radiological and nuclear disasters

Ionizing radiation is responsible for the production of free radicals, including reactive oxygen species and reactive nitrogen species. When bone marrow or gastrointestinal systems receive an acute high dose of radiation (typically more than 4 Gy), exposed people can die as a result of acute radiation syndrome [22]. However, studies have shown that the exposure of other organs, such as the lung and heart, to radiation resulting from inhaling radionuclides or external exposure can cause acute reactions, leading to the inactivation of these organs and death after a number of months [23, 24]. In addition to the risk of death after a radiological or nuclear disaster, many people exposed to radiation-contaminated areas show signs of disease in years long after exposure [25, 26].

Hundreds of radiological and nuclear events have occurred since the World War II [27]. The nuclear disaster in Hiroshima and Nagasaki during the World War II is the most important example of the importance of radiation toxicity in the development of various types of diseases. In this disaster, more than 150,000 people immediately died, while thousands of people received sub-lethal doses [28]. Subsequently, the Chernobyl disaster was the most important environmental nuclear disaster [29]. Epidemiological studies performed some decades after the nuclear disaster have confirmed chronic changes in several biological factors, particularly the factors related to cancer and the immune system [30]. Increased incidences of thyroid diseases resulting from radioactive iodine was one of the most common diseases in some adjacent countries to Chernobyl [31]. The incidence of cancer and increased level of inflammatory markers have been reported among Hiroshima and Nagasaki survivors [32]. The chronic inflammation induced by IR as a result of changes in adaptive and innate immune responses is responsible for various disorders among exposed people. These disorders include cardiovascular diseases, diabetes, and damage to thyroid function [33, 34].

## Innate and adaptive immune responses: Mechanisms of autoimmunity diseases

The innate immune system provides the host with an immediate response against pathogens. This system senses pathogens and damaged cells through damage associated molecular patterns (DAMPs) by pattern recognition receptors (PRRs). PRRs are ligands primarily expressed by innate immune system cells, such as macrophages, T-cells, and dendritic cells (DCs) [35]. The most important type of PRRs is toll-like receptors (TLRs). TLRs detect a variety of DAMPs, such as oxidized DNA, high-mobility group box 1 (HMGB1), and uric acid [36, 37]. Studies have proposed that TLR2, TLR4, TLR5, TLR7, and TLR9 are the main PRRs, which stimulate inflammatory responses following the exposure of innate immune cells to pathogens or damaged cells [38, 39]. These TLRs, through the stimulation of myeloid differentiation primary response 88 (MyD88), induce the upregulation NF-κB and other transcription factors, leading to the secretion of pro-inflammatory cytokines [40]. For example, the ligation of DAMPs to TLR7 and TLR9 can stimulate the secretion of IFN-γ from inflammatory cells, while TLR2 and TLR4 can upregulate the secretion of IL-1β, IL-6, and IL-8 [41].

Another part of the immune system includes the adaptive immune system mediated by B and T cells. The B cells mediate humoral immunity, while T cells mediate cellular immunity. These cells distinguish self-antigens from foreign antigens. The B cell receptors (BCRs) and T cell receptors (TCRs), which are generated through VDJ recombination, are responsible for the recognition of antigens [42]. However, antigen-presenting cells (APCs), including DCs, B-cells, and macrophages, present antigens to these cells. The interaction of these immune cells is necessary for the detection and response of the adaptive immune system. Presenting antigens by APCs is associated with the activation of T cells, including CD8^+^ cytotoxic T-cells (CTL) or CD4^+^T helper cells [43, 44].

Typically, autoimmune diseases result from altering the T cell-dependent control of self-reactive T cells. Abnormal changes in the levels of some cytokines and activities of APCs can result in the abnormal activation of CTLs. DCs play a key role in presenting danger alarms to T cells, leading to the reaction of these cells [45]. Studies have shown that increasing the level of some cytokines, such as IL-1, TNF-α and IFN-γ, lead to alterations in DCs homeostasis. These cytokines may induce the differentiation of immature DCs into mature DCs. The increased differentiation of immature DCs has been associated with various autoimmune diseases. [46]. IL-6 is another cytokine involved in the pathogenesis of autoimmune diseases [47, 48]. Blocking these cytokines by their antagonists and using anti-inflammatory cytokines, such as TGF-β1, can reduce the severity of autoimmune processes [49, 50]. These results indicated that there is a strong link between chronic inflammation after exposure to IR and autoimmune disorders.

## Radiation-induced inflammation

Inflammation is one of the most important responses of tissues to IR, which can cause damage to various organs for years after exposure. Inflammation is a complicated process that appears as damage of the vasculature, migration of leukocytes into the irradiated area and the release of various immune system mediators [51, 52]. The responses of normal tissues to IR are highly dependent on the radiation dose. With increasing radiation dose, the incidence of vascular damage, hypoxia and cell necrosis increases. This effect is associated with changes in the immune system response, leading to changes in the cytokine profile. The exposure of body cells to low doses of IR (lower than 1 Gy) can stimulate anti-inflammatory effects. This effect results from the high incidence of apoptosis compared to necrosis, while exposure to higher doses of IR (more than 1 Gy) leads to necrosis, rather than apoptosis, causing inflammation responses [18, 53].

Although vascular damage and necrosis are responsible for the initiation of inflammation, under stress conditions, such as exposure to a heavy dose of IR, other types of cell death, such as apoptosis, autophagy, and senescence, can stimulate inflammatory responses [54]. Massive DNA damage and cell death following exposure to high doses of IR (more than 1 Gy) lead to the release of cellular contents, such as danger alarms or DAMPs. The most important released DAMPs after exposure to IR include HMGB1, uric acid, and heat-shock proteins (HSPs). In response to IR, TLR2, TLR4, TLR5 and TLR9 play a central role in the activation of inflammation pathways via the identification of DAMPs [54, 55].

**Fig. 1:**
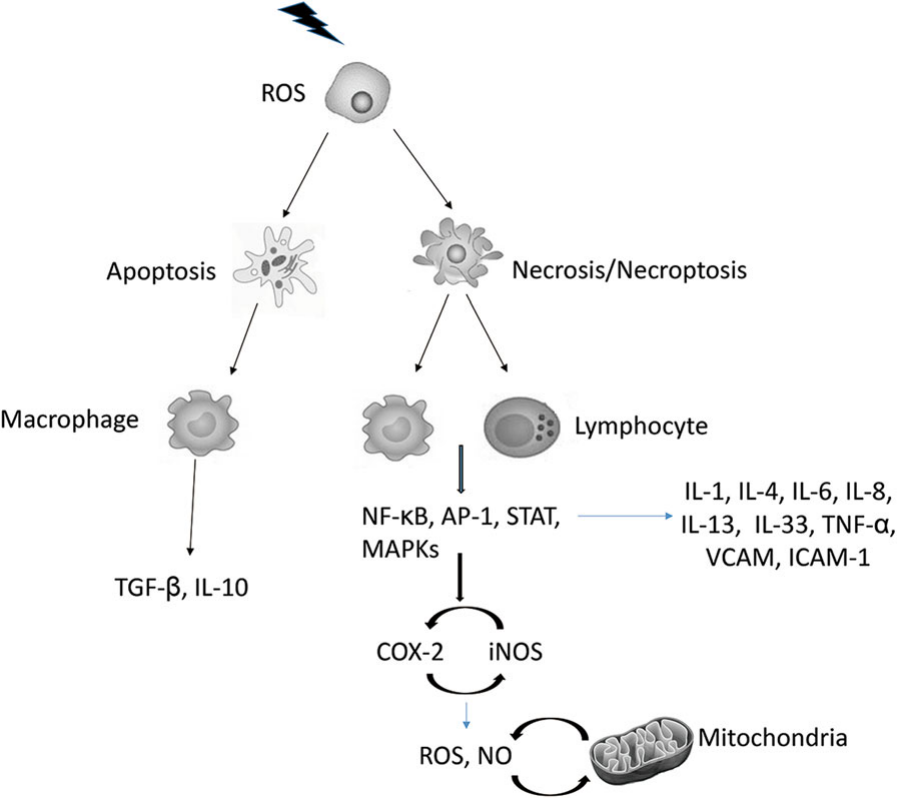
The mechanisms of IR-induced inflammation or anti-inflammatory responses. After apoptosis, macrophages detect apoptotic bodies, leading to the secretion of anti-inflammatory cytokines, such as transforming growth factor beta (TGF-β). This response is observed for low doses of ionizing radiation (IR) (less than 1 Gy), where apoptosis is more common than necrosis. However, damage associated molecular patterns (DAMPs) released by necrotic cells after exposure to high doses of IR stimulate T-cells and dendritic cells (DCs) to upregulate inflammatory mediators and the secretion of inflammatory cytokines

TLRs through the upregulation of inflammatory mediators, such as MAPKs, NF-κB, and COX-2, trigger the secretion of inflammatory cytokines, including IL-1, IL-6, IL-8, TNF, IL-33, and IFN-γ. However, these cytokines further amplify the regulation of inflammatory mediators in a positive feedback loop [56]. In addition to inflammatory mediators, continuous ROS and nitric oxide production amplify the toxic effects of radiation-induced inflammation on normal tissues. When this response is not inhibited by anti-inflammatory mechanisms, the inflammatory cytokines and free radicals induced by chronic inflammation disrupt the normal function of organs [56, 57] (Fig. 1).

## Evidence of IR-induced autoimmune diseases

Epidemiological studies of the atomic-bomb survivors have recommended that IR can functionally break self-tolerance and induce various organ-specific autoimmune diseases [30, 58]. Impaired DNA damage responses are postulated as key mechanisms involved in some autoimmune diseases, such as type I diabetes, rheumatoid arthritis, gastritis, thyroiditis, and orchitis [59]. An in-vivo study showed that induction of autoimmune diseases occurs following the irradiation of both the thymus and peripheral lymphoid organs [60]. However, there is clear evidence that lymphocytes from patients with autoimmune diseases, such as systemic sclerosis and rheumatoid arthritis, exhibit higher radiosensitivity and delayed DNA damage repair responses compared to lymphocytes from healthy donors [61, 62]. Thus, autoimmune diseases induced by radiation may be involved in carcinogenesis in years long after exposure in the irradiated population. To the best of our knowledge, thyroid diseases and diabetes are two important autoimmune diseases linked to radiation exposure in the population.

### Thyroid autoimmune diseases

There are several reports linking autoimmune thyroid disease to therapeutic and environmental radiation exposures. Examples for this claim include Japanese atomic bomb survivors, children exposed to radioactive iodine from ^131^I therapy and nuclear weapons testing at the Nevada test site, and Chernobyl disaster survivors. Several studies have reported abnormal incidences of autoimmune reactions in the pathogenesis of thyroid diseases and impaired natural killer (NK) cells activity against tumor cells [63–68]. Additionally, occupational exposures to IR among nuclear power plant, medical and laboratory workers are related to a higher risk of autoimmune reactions [69]. A study on Hiroshima and Nagasaki atomic bomb survivors and children exposed to radioactive iodine from nuclear weapons testing showed a linear radiation dose response for thyroid tumors and nodules but not for autoimmune thyroid diseases [66, 70]. However, the increased risk of autoimmune thyroid diseases may have contributed to the elevated cancer incidence among the irradiated population [70].

In addition to high doses of IR in radiation therapy or disasters, several studies have proposed that exposure to occupational doses of IR may be associated with thyroid autoimmune disease. In a cross-sectional study, Völzke et al. [66] examined the potential role of the low-dose occupational exposures to IR (mostly lower than 1–2 mSV in a year) associated with the increased risk of thyroid diseases. These authors used thyroid ultrasound and antithyroxiperoxidase antibodies to detect the incidence of this disease. The results showed an unusual thyroid pattern in ultrasound imaging, particularly for females. Moreover, the results showed that this risk is higher for workers of a nuclear power plant, particularly those that have been exposed for more than 5 years [71].

Imaizumi and colleagues evaluated the risk of thyroid diseases among atomic bomb survivors exposed in utero. Among the 319 participants that received an equal radiation dose of 25 cGy, no significant thyroid disease was detected. However, the authors proposed that a limited statistical power may be involved in the negative result of this study [72]. As the radiosensitivity of the uterus is similar to that of the child, further studies with a higher population number are needed to investigate the potential role of IR in thyroid diseases in utero.

### Potential mechanisms of IR-induced thyroid autoimmune diseases

Studies have proposed that autoimmune thyroid disease is related to genetic background, while exposure to toxic agents, such as ionizing radiation, is also involved [73, 74]. Mechanisms of autoimmune thyroid diseases are different, which can be observed as changes in thyroid hormone action, resulting in a change in circulating hormones. However, thyroid malfunctions may result from pituitary and thyrotropin secretion [75].

The most common thyroid diseases following exposure to ionizing radiation are hypofunction and thyroid nodules. However, the increased incidence of thyroid cancers may be involved in these diseases [76]. A reduction in function of the thyroid is the most common autoimmune disease induced by IR. Graves’ disease has been implicated in hypothyroidism or hyperthyroidism following exposure. In addition, a reduction of the thyroid-stimulating hormone(TSH) receptor in patients treated with radioiodine is associated with the increased probability of autoimmune hyperthyroidism [77].

Several studies have proposed that inflammatory responses may be involved in autoimmune diseases. For example, the production of inflammatory cytokines, such as IL-1 and IL-6, in thyroid-infiltrating mononuclear and follicular cells has been associated with Graves’ disease [78]. Macrophages and NK cells play a central role in the secretion of IL-1 and IL-6. IL-1 can stimulate the release of IL-6 in thyrocytes. Moreover, this effect may be potentiated by TNF-α. The upregulation of these inflammatory cytokines can suppress the secretion of thyroid hormones, such as TSH, and reduce the serum levels of Thyroxine(T4) and Triiodothyronine (T3) [79, 80].

### Diabetes

Several studies have reported a link between whole body or abdomen irradiation and the increased risk of diabetes [81–83]. A previous study showed a two-fold increased risk of diabetes mellitus (especially type 2) for cancer survivors treated by radiotherapy for acute myeloid leukemia (AML), neuroblastoma, Wilms tumor, and Hodgkin lymphoma. Additionally, survivors of acute lymphoblastic leukemia (ALL) and brain tumors showed an increased risk of obesity in adulthood. The results of this study showed that neuroblastoma survivors have the highest likelihood of becoming diabetic compared with siblings. This issue is more obvious for individuals who have been exposed to whole body irradiation and abdomen radiotherapy for childhood cancer [84, 85]. The association between Hodgkin lymphoma treatment and the increased risk of diabetes mellitus has been confirmed [86]. In a 30-year follow-up study of 2520 childhood cancer survivors, Vathaire et al. [83] showed that irradiation to the tail of the pancreas is closely associated with the increased risk of diabetes. This study showed that the risk of diabetes has a strong relationship with radiation dose. The irradiation of other parts of the pancreas did not show any relationship with diabetes risk [87]. The mechanisms of IR-induced diabetes are not fully understood. However, the impairment of specific β-cell and insulin release among cancer survivors may be involved [88].

### Potential mechanisms of IR-induced diabetes

In contrast to the aforementioned reports, several studies have indicated that low-dose radiation (0.5 Gy) has a prevention effect on the development of diabetes. Mechanisms may include the induction of pancreatic antioxidants and protection of the β cells from oxidative damage and immunomodulation to preserve pancreatic function [89]. However, the precise molecular mechanism of β-cell damage after irradiation is not known, and chronic inflammation likely plays a key role in this process. It is feasible that pro-inflammatory cytokines, such as IL-1β and IFN-γ, be involved in the signaling pathways that cause pancreatic β-cell death and dysfunction [90]. Moreover, activation of the NLRP3 inflammasome may stimulate oxidative damage through hyperglycemia [91–93]. However, there is some evidence that the proposed genotype is involved in the risk of radiation-induced diabetes. A study of atomic-bomb survivors showed a relationship between human leukocyte antigen (HLA) genotypes and diabetes. Survivors with the DQA1*03-DRB1*09 or DQA1*0401-DRB1*08 haplotypes showed an increased risk of diabetes with radiation dose [94].

### Gastritis

**Fig. 2:**
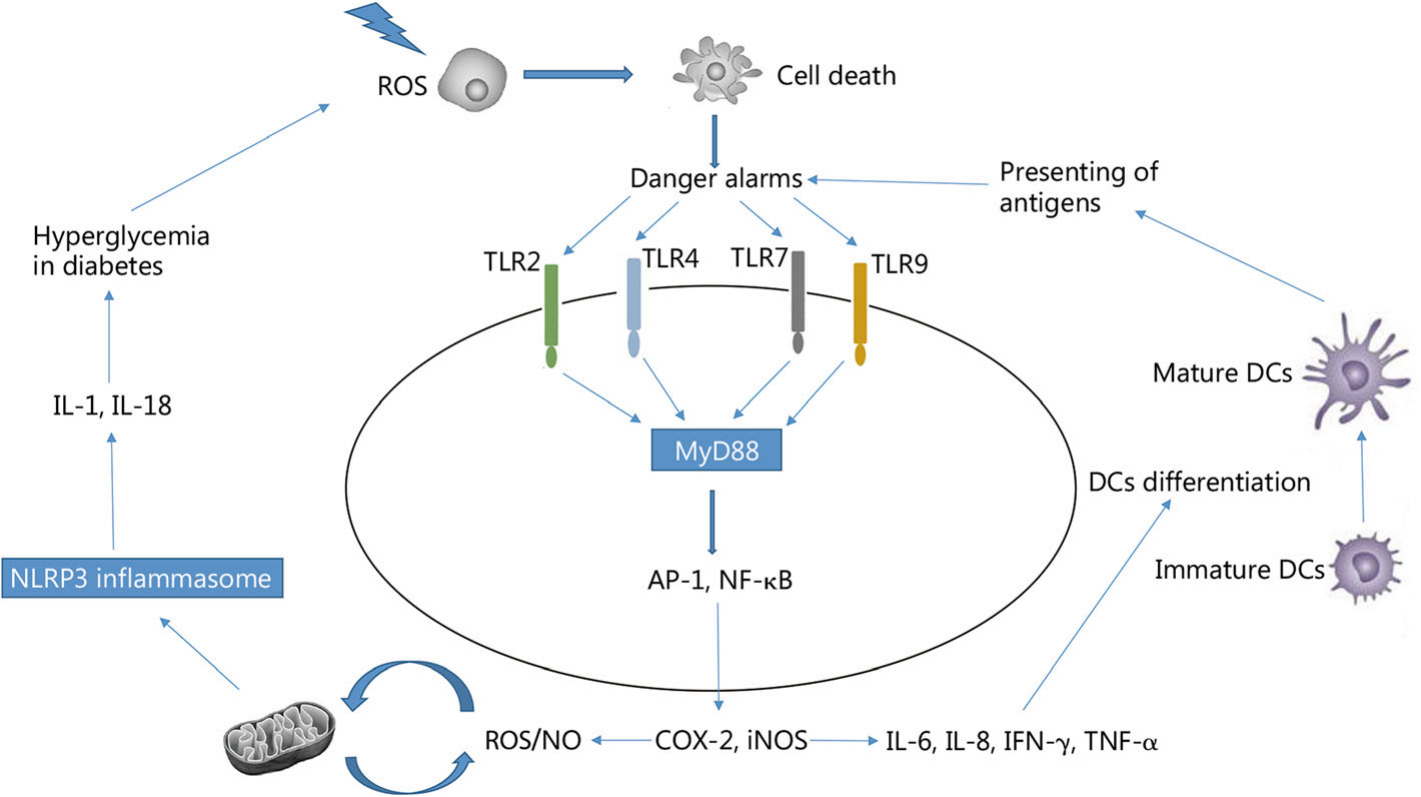
Model for radiation-induced inflammatory autoimmune diseases. In this model, ionizing radiation (IR) induces reactive oxygen species (ROS) production and necrosis, leading to the activation of inflammatory transcription factors and secretion of inflammatory cytokines. This effect is associated with further ROS production and cell death. The activation of the inflammasome in diabetic patients is involved in hyperglycemia, leading to oxidative damage. Moreover, inflammatory cytokines can stimulate the differentiation of dendritic cells (DCs), which present further necrosis antigens to T-cells

Autoimmune gastritis is a chronic inflammatory gastric condition characterized by the increased number and activity of inflammatory cells, such as lymphocytes. Chronic gastritis results from the destruction of the mucosa, leading to pain, atrophy, inflammation and bleeding [95]. This type of autoimmune disease is common for clinical tumor treatment in which the stomach is within the field of radiotherapy. Gastritis typically appears after the gastric exposure to doses higher than 40–50 Gy [96]. Although the molecular mechanisms of gastritis following exposure to radiation have not been completely determined, several studies have reported that the suppression of inflammation using anti-inflammatory drugs can help control this condition [97–99]. Graziani et al. proposed that radiation-induced gastritis in patients with pancreatic cancer can be stimulated by other drugs, such as erlotinib, which is an antagonist for the expression of epidermal growth factor receptor [100] (Fig. 2).

## Potential role of radiation mitigators to prevent radiation-induced inflammation diseases

Although the administration of antioxidants or other radiation modifiers prior to exposure has the highest efficiency, several studies have proposed that chronic treatment with antioxidant agents may reduce the long-term consequences of radiation. For example, the treatment of Chernobyl survivors with antioxidants, such as beta-carotene, reduced the serum level of oxidative damage markers [101]. As previously described, several studies have confirmed that chronic inflammation plays a key role in the detrimental effects of radiation, including continuous oxidative stress and even carcinogenesis. Treatment with some antioxidants, such as ascorbic acid, selenium, and other antioxidant mixtures, have exhibited the ability to ameliorate increased oxidative damage and the levels of inflammatory cytokines, such as IL-1, IL-6, TNF-α, IFN-γ, and TGF-β [102–104]. In vivo studies have confirmed the mitigatory effects of certain antioxidants and immunomodulators for other radiation-induced inflammation diseases, such as nephrotoxicity, pneumonitis and fibrosis [105–109]. As autoimmune diseases have a direct relation to these factors, it is likely that the amelioration of chronic inflammation may help reduce the incidence of various radiation-induced diseases, including autoimmune diseases.

## Conclusions

Although there is a long way to illustrate the complete mechanisms of IR-induced diabetes and thyroid diseases, recent studies suggest that the chronic upregulation of inflammatory mediators and cytokines play a key role. It is feasible that long-term exposure to a high level of some cytokines, particularly IL-1β and IFN-γ, can disrupt the normal function of thyrocytes in the thyroid, as well as beta cells in the pancreas. However, it is likely that genetic background plays a key role in the appearance of autoimmune diseases. Although there is no suggested strategy for the prevention or reduction of autoimmune diseases following exposure to IR during radiotherapy or radiation accidents, there may be several solutions, such as the administration of radioprotectors, anti-inflammatory agents, or radiation mitigators. However, to date, there is no evidence to confirm this idea.

## Abbreviations

AP-1: Activator protein 1
COX-2: Cyclooxygenase-2
DCs: Dendritic cells
HMGB1: High-mobility group box 1
HSPs: Heat-shock proteins
ICAM-1: Intercellular adhesion molecule 1
IFN-γ: Interferon gamma
IGF-1: Insulin-like growth factor 1
IL: Interleukin
iNOS: Inducible nitric oxide synthase
IR: Ionizing radiation
MAPKs: Mitogen-activated protein kinases
NADPH: Nicotinamide adenine dinucleotide phosphate
NF- κB: Nuclear factor- κB
NO: Nitric oxide
ROS: Reactive oxygen species
STAT: Signal transducers and activators of transcription
T3: Triiodothyronine
T4: Thyroxine
T-Cells: Lymphocytes T
TGF-β: Transforming growth factor beta
TLR: Toll-like receptor
TNF-α: Tumor necrosis factor
TSH: Thyroid-stimulating hormone
VCAM: Vascular cell adhesion molecule 1

## References

[1] Wong FL, Yamada M, Sasaki H, Kodama K, Akiba S, Shimaoka K (1993). Noncancer disease incidence in the atomic bomb survivors: 1958-1986. Radiat Res.

[2] Rezapoor S, Shirazi A, Abbasi S, Bazzaz JT, Izadi P, Rezaeejam H (2017). Modulation of radiation-induced base excision repair pathway gene expression by melatonin. J Med Phys.

[3] Georgakilas AG, Pavlopoulou A, Louka M, Nikitaki Z, Vorgias CE, Bagos PG (2015). Emerging molecular networks common in ionizing radiation, immune and inflammatory responses by employing bioinformatics approaches. Cancer Lett.

[4] Wu Y, Antony S, Meitzler JL, Doroshow JH (2014). Molecular mechanisms underlying chronic inflammation-associated cancers. Cancer Lett.

[5] Hasselbalch HC (2013). Chronic inflammation as a promotor of mutagenesis in essential thrombocythemia, polycythemia vera and myelofibrosis. A human inflammation model for cancer development?. Leuk Res.

[6] Roder DM (2002). The epidemiology of gastric cancer. Gastric Cancer.

[7] New DD, Block K, Bhandhari B, Gorin Y, Abboud HE (2012). IGF-I increases the expression of fibronectin by Nox4-dependent Akt phosphorylation in renal tubular epithelial cells. Am J Physiol Cell Physiol.

[8] Sedeek M, Montezano AC, Hebert RL, Gray SP, Di Marco E, Jha JC (2012). Oxidative stress, Nox isoforms and complications of diabetes—potential targets for novel therapies. J Cardiovasc Transl Res.

[9] Rezaie A, Parker RD, Abdollahi M (2007). Oxidative stress and pathogenesis of inflammatory bowel disease: an epiphenomenon or the cause?. Dig Dis Sci.

[10] Tak PP, Zvaifler NJ, Green DR, Firestein GS (2000). Rheumatoid arthritis and p53: how oxidative stress might alter the course of inflammatory diseases. Immunol Today.

[11] Leung PS, Chan YC (2009). Role of oxidative stress in pancreatic inflammation. Antioxid Redox Signal.

[12] Bullon P, Newman HN, Battino M (2014). Obesity, diabetes mellitus, atherosclerosis and chronic periodontitis: a shared pathology *via* oxidative stress and mitochondrial dysfunction?. Periodontol.

[13] Tucker PS, Scanlan AT, Dalbo VJ (2015). Chronic kidney disease influences multiple systems: describing the relationship between oxidative stress, inflammation, kidney damage, and concomitant disease. Oxidative Med Cell Longev.

[14] Einor D, Bonisoli-Alquati A, Costantini D, Mousseau T, Møller A (2016). Ionizing radiation, antioxidant response and oxidative damage: a meta-analysis. Sci Total Environ.

[15] Lushchak VI (2014). Free radicals, reactive oxygen species, oxidative stress and its classification. Chem Biol Interact.

[16] Nimse SB, Pal D (2015). Free radicals, natural antioxidants, and their reaction mechanisms. RSC Adv.

[17] Najafi M, Shirazi A, Motevaseli E, Geraily G, Norouzi F, Heidari M, Rezapoor S (2017). The melatonin immunomodulatory actions in radiotherapy. Biophys Rev.

[18] Najafi M, Shirazi A, Motevaseli E, Rezaeyan AH, Salajegheh A, Rezapoor S (2017). Melatonin as an anti-inflammatory agent in radiotherapy. Inflammopharmacology.

[19] Fardid RSA, Mosleh-Shirazi MA, Sharifzadeh S, Okhovat MA, Najafi M, Rezaeyan A (2017). Melatonin ameliorates the production of COX-2, iNOS, and the formation of 8-OHdG in non-targeted lung tissue after pelvic irradiation. Cell J.

[20] Schaue D, Micewicz ED, Ratikan JA, Xie MW, Cheng G, McBride WH (2015). Radiation and inflammation. Semin Radiat Oncol.

[21] Zhao W, Robbins ME (2009). Inflammation and chronic oxidative stress in radiation-induced late normal tissue injury: therapeutic implications. Curr Med Chem.

[22] Macià i, Garau M, Lucas Calduch A, López EC (2011). Radiobiology of the acute radiation syndrome. Rep Pract Oncol Radiother.

[23] Medhora M, Gao F, Jacobs ER, Moulder JE (2012). Radiation damage to the lung: mitigation by angiotensin-converting enzyme (ACE) inhibitors. Respirology.

[24] Najafi M, Motevaseli E, Shirazi A, Graily G, Rezaeyan A, Norouzi F, Rezapoor S, Abdollahi H. Mechanisms of inflammatory responses to radiation and normal tissues toxicity; clinical implications. Int J Radiat Biol. 2018. 10.1080/09553002.2018.1440092. 10.1080/09553002.2018.1440092 29504497

[25] Ivanov VK, Maksioutov MA, Chekin SY, Petrov AV, Biryukov AP, Kruglova ZG (2006). The risk of radiation-induced cerebrovascular disease in Chernobyl emergency workers. Health Phys.

[26] Boice JD (2006). Thyroid disease 60 years after Hiroshima and 20 years after Chernobyl. JAMA.

[27] Hecht G, Callon M (2009). The radiance of France: nuclear power and national identity after world war II (inside technology).

[28] Shimizu Y, Kodama K, Nishi N, Kasagi F, Suyama A, Soda M (2010). Radiation exposure and circulatory disease risk: Hiroshima and Nagasaki atomic bomb survivor data, 1950-2003. BMJ.

[29] Berger EM (2010). The Chernobyl disaster, concern about the environment, and life satisfaction. Kyklos.

[30] Kusunoki Y, Hayashi T (2008). Long-lasting alterations of the immune system by ionizing radiation exposure: implications for disease development among atomic bomb survivors. Int J Radiat Biol.

[31] Detours V, Delys L, Libert F, Solis DW, Bogdanova T, Dumont JE (2007). Genome-wide gene expression profiling suggests distinct radiation susceptibilities in sporadic and post-Chernobyl papillary thyroid cancers. Br J Cancer.

[32] McGregor DH, Land C, Choi K, Tokuoka S, Liu PI, Wakabayashi T (1977). Breast cancer incidence among atomic bomb survivors, Hiroshima and Nagasaki, 1950–69. J Natl Cancer Inst.

[33] Belsky JL, Tachikawa K, Jablon S (1973). The health of atomic bomb survivors: a decade of examinations in a fixed population. Yale J Biol Med.

[34] Douple EB, Mabuchi K, Cullings HM, Preston DL, Kodama K, Shimizu Y (2011). Long-term radiation-related health effects in a unique human population: lessons learned from the atomic bomb survivors of Hiroshima and Nagasaki. Disaster Med Public Health Prep.

[35] McCarthy CG, Goulopoulou S, Wenceslau CF, Spitler K, Matsumoto T, Webb RC (2014). Toll-like receptors and damage-associated molecular patterns: novel links between inflammation and hypertension. Am J Physiol Heart Circ Physiol.

[36] Tang D, Kang R, Zeh HJ, Lotze MT (2011). High-mobility group box 1, oxidative stress, and disease. Antioxid Redox Signal.

[37] Yang H, Antoine DJ, Andersson U, Tracey KJ (2013). The many faces of HMGB1: molecular structure-functional activity in inflammation, apoptosis, and chemotaxis. J Leukoc Biol.

[38] Akira S, Takeda K, Kaisho T (2001). Toll-like receptors: critical proteins linking innate and acquired immunity. Nat Immunol.

[39] Takeuchi O, Akira S (2010). Pattern recognition receptors and inflammation. Cell.

[40] Verstak B, Nagpal K, Bottomley SP, Golenbock DT, Hertzog PJ, Mansell A (2009). MyD88 adapter-like (mal)/TIRAP interaction with TRAF6 is critical for TLR2-and TLR4-mediated NF-κB proinflammatory responses. J Biol Chem.

[41] Tang L, Zhou XD, Wang Q, Zhang L, Wang Y, Li XY (2011). Expression of TRAF6 and pro-inflammatory cytokines through activation of TLR2, TLR4, NOD1, and NOD2 in human periodontal ligament fibroblasts. Arch Oral Biol.

[42] Delves PJ, Roitt IM (2000). The immune system. N Engl J Med.

[43] Barinov A, Galgano A, Krenn G, Tanchot C, Vasseur F, Rocha B (2017). CD4/CD8/dendritic cell complexes in the spleen: CD8+ T cells can directly bind CD4+ T cells and modulate their response. PLoS One.

[44] Kurts C, Heath WR, Carbone FR, Kosaka H, Miller JF (1998). Cross-presentation of self antigens to CD8+ T cells: the balance between tolerance and autoimmunity. Novartis Found Symp.

[45] Gallo PM, Gallucci S. The dendritic cell response to classic, emerging, and homeostatic danger signals. Implications for autoimmunity. Front Immunol. 2013;4(138) 10.3389/fimmu.2013.00138 PMC3677085 23772226

[46] Blanco P, Palucka AK, Pascual V, Banchereau J (2008). Dendritic cells and cytokines in human inflammatory and autoimmune diseases. Cytokine Growth Factor Rev.

[47] Ishihara K, Hirano T (2002). IL-6 in autoimmune disease and chronic inflammatory proliferative disease. Cytokine Growth Factor Rev.

[48] Neurath MF, Finotto S (2011). IL-6 signaling in autoimmunity, chronic inflammation and inflammation-associated cancer. Cytokine Growth Factor Rev.

[49] Dinarello C (1992). Interleukin-1 and tumor necrosis factor: effector cytokines in autoimmune diseases. Semin Immunol.

[50] Prud'Homme GJ, Piccirillo CA (2000). The inhibitory effects of transforming growth factor-beta-1 (TGF-β1) in autoimmune diseases. J Autoimmun.

[51] Haddadi GH, Rezaeyan A, Mosleh-Shirazi MA, Hosseinzadeh M, Fardid R, Najafi M (2017). Hesperidin as radioprotector against radiation-induced lung damage in rat: a histopathological study. J Med Phys..

[52] Yahyapour R, Motevaseli E, Rezaeyan A, Abdollahi H, Farhood B, Cheki M, et al. Mechanisms of radiation bystander and non-targeted effects: implications to radiation carcinogenesis and radiotherapy. Curr Radiopharm. 2017;11(1):34–45. 10.2174/1874471011666171229123130 29284398

[53] Rodel F, Frey B, Gaipl U, Keilholz L, Fournier C, Manda K (2012). Modulation of inflammatory immune reactions by low-dose ionizing radiation: molecular mechanisms and clinical application. Curr Med Chem.

[54] Multhoff G, Radons J (2012). Radiation, inflammation, and immune responses in cancer. Front Oncol.

[55] Yahyapour R, Motevaseli E, Rezaeyan A, Abdollahi H, Farhood B, Cheki M, et al. Reduction-oxidation (redox) system in radiation-induced normal tissue injury: molecular mechanisms and implications in radiation therapeutics. Clin Transl Oncol. 2018. 10.1007/s12094-017-1828-6. PMID: 29318449. 10.1007/s12094-017-1828-6 29318449

[56] Multhoff G, Molls M, Radons J (2011). Chronic inflammation in cancer development. Front Immunol.

[57] Mavragani IV, Nikitaki Z, Souli MP, Aziz A, Nowsheen S, Aziz K (2017). Complex DNA damage: a route to radiation-induced genomic instability and carcinogenesis. Cancers.

[58] Akiyama M (1995). Late effects of radiation on the human immune system: an overview of immune response among the atomic-bomb survivors. Int J Radiat Biol.

[59] Bhusate L, Herbert K, Scott D, Perrett D (1992). Increased DNA strand breaks in mononuclear cells from patients with rheumatoid arthritis. Ann Rheum Dis.

[60] Sakaguchi N, Miyai K, Sakaguchi S (1994). Ionizing radiation and autoimmunity. Induction of autoimmune disease in mice by high dose fractionated total lymphoid irradiation and its prevention by inoculating normal T cells. J Immunol.

[61] McCurdy D, Tai L, Frias S, Wang Z (1997). Delayed repair of DNA damage by ionizing radiation in cells from patients with juvenile systemic lupus erythematosus and rheumatoid arthritis. Radiat Res.

[62] Harris G, Cramp W, Edwards JC, George A, Sabovljev S, Hart L (1985). Radiosensitivity of peripheral blood lymphocytes in autoimmune disease. Int J Radiat Biol.

[63] Sklar C, Whitton J, Mertens A, Stovall M, Green D, Marina N (2000). Abnormalities of the thyroid in survivors of Hodgkin’s disease: data from the childhood cancer survivor study 1. J Clin Endocrinol Metab.

[64] Nagataki S, Shibata Y, Inoue S, Yokoyama N, Izumi M, Shimaoka K (1994). Thyroid diseases among atomic bomb survivors in Nagasaki. JAMA.

[65] Agate L, Mariotti S, Elisei R, Mossa P, Pacini F, Molinaro E (2008). Thyroid autoantibodies and thyroid function in subjects exposed to Chernobyl fallout during childhood: evidence for a transient radiation-induced elevation of serum thyroid antibodies without an increase in thyroid autoimmune disease. J Clin Endocrinol Metab.

[66] Lyon JL, Alder SC, Stone MB, Scholl A, Reading JC, Holubkov R (2006). Thyroid disease associated with exposure to the Nevada nuclear weapons test site radiation: a reevaluation based on corrected dosimetry and examination data. Epidemiology.

[67] Soule J, Mayfield R (2001). Graves' disease after 131I therapy for toxic nodule. Thyroid.

[68] Dunkelmann S, Wolf R, Koch A, Kittner C, Groth P, Schuemichen C (2004). Incidence of radiation-induced graves’ disease in patients treated with radioiodine for thyroid autonomy before and after introduction of a high-sensitivity TSH receptor antibody assay. Eur J Nucl Med Mol Imaging.

[69] Völzke H, Werner A, Wallaschofski H, Friedrich N, Robinson DM, Kindler S (2005). Occupational exposure to ionizing radiation is associated with autoimmune thyroid disease. J Clin Endocrinol Metab.

[70] Imaizumi M, Tominaga T, Neriishi K, Akahoshi M, Nakashima E, Ashizawa K (2006). Radiation dose-response relationships for thyroid nodules and autoimmune thyroid diseases in Hiroshima and Nagasaki atomic bomb survivors 55-58 years after radiation exposure. JAMA.

[71] Sinnott B, Ron E, Schneider AB. Exposing the Thyroid to Radiation: A Review of Its Current Extent, Risks, and Implications. Endocrine Reviews. 2010;31(5):756–73. 10.1210/er.2010-0003 PMC3365850 20650861

[72] Imaizumi M, Ashizawa K, Neriishi K, Akahoshi M, Nakashima E, Usa T (2008). Thyroid diseases in atomic bomb survivors exposed in utero. J Clin Endocrinol Metab.

[73] Tomer Y, Huber A (2009). The etiology of autoimmune thyroid disease: a story of genes and environment. J Autoimmun.

[74] Jacobson EM, Tomer Y (2007). The genetic basis of thyroid autoimmunity. Thyroid.

[75] Brent GA (2010). Environmental exposures and autoimmune thyroid disease. Thyroid.

[76] Feldt-Rasmussen U, Rasmussen AK (2010). Autoimmunity in differentiated thyroid cancer: significance and related clinical problems. Hormones (Athens).

[77] Ma J-A, Li X, Zou W, Zhou Y. Grave’s disease induced by radiotherapy for nasopharyngeal carcinoma: A case report and review of the literature. Oncology Letters. 2013;6(1):144–46. 10.3892/ol.2013.1332 PMC3742527 23946792

[78] Grubeck-Loebenstein B, Buchan G, Chantry D, Kassal H, Londei M, Pirich K (1989). Analysis of intrathyroidal cytokine production in thyroid autoimmune disease: thyroid follicular cells produce interleukin-1 alpha and interleukin-6. Clin Exp Immunol.

[79] Fujii T, Sato K, Ozawa M, Kasono K, Imamura H, Kanaji Y (1989). Effect of interleukin-1 (IL-1) on thyroid hormone metabolism in mice: stimulation by IL-1 of iodothyronine 5′-deiodinating activity (type I) in the liver. Endocrinology.

[80] Bendtzen K, Buschard K, Diamant M, Horn T, Svenson M (1989). Possible role of IL-1, TNF-alpha, and IL-6 in insulin-dependent diabetes mellitus and autoimmune thyroid disease. Thyroid cell group. Lymphokine Res.

[81] Ito C (1994). Trends in the prevalence of diabetes mellitus among Hiroshima atomic bomb survivors. Diabetes Res Clin Pract.

[82] Hoffmeister PA, Storer BE, Sanders JE (2004). Diabetes mellitus in long-term survivors of pediatric hematopoietic cell transplantation. J Pediatr Hematol Oncol.

[83] Baker KS, Ness KK, Steinberger J, Carter A, Francisco L, Burns LJ (2007). Diabetes, hypertension, and cardiovascular events in survivors of hematopoietic cell transplantation: a report from the bone marrow transplantation survivor study. Blood.

[84] Meacham LR, Sklar CA, Li S, Liu Q, Gimpel N, Yasui Y (2009). Diabetes mellitus in long-term survivors of childhood cancer: increased risk associated with radiation therapy: a report for the childhood cancer survivor study. Arch Intern Med.

[85] Najafi M, Cheki M, Rezapoor S, Geraily G, Motevaseli E, Carnovale C (2018). Metformin: prevention of genomic instability and cancer: a review. Mutat Res Genet Toxicol Environ Mutagen.

[86] Van Nimwegen FA, Schaapveld M, Janus CP, Krol AD, Raemaekers JM, Kremer LC (2014). Risk of diabetes mellitus in long-term survivors of Hodgkin lymphoma. J Clin Oncol.

[87] de Vathaire F, El-Fayech C, Ayed FFB, Haddy N, Guibout C, Winter D (2012). Radiation dose to the pancreas and risk of diabetes mellitus in childhood cancer survivors: a retrospective cohort study. Lancet Oncol.

[88] Wei C, Thyagiarajan M, Hunt L, Cox R, Bradley K, Elson R (2015). Reduced beta-cell reserve and pancreatic volume in survivors of childhood acute lymphoblastic leukaemia treated with bone marrow transplantation and total body irradiation. Clin Endocrinol.

[89] Wang GJ, Li XK, Sakai K, Cai L (2008). Low-dose radiation and its clinical implications: diabetes. Hum Exp Toxicol.

[90] Collier JJ, Burke SJ, Eisenhauer ME, Lu D, Sapp RC, Frydman CJ (2011). Pancreatic beta-cell death in response to pro-inflammatory cytokines is distinct from genuine apoptosis. PLoS One.

[91] Oslowski CM, Hara T, O'Sullivan-Murphy B, Kanekura K, Lu S, Hara M (2012). Thioredoxin-interacting protein mediates ER stress-induced β cell death through initiation of the inflammasome. Cell Metab.

[92] Zhou R, Tardivel A, Thorens B, Choi I, Tschopp J (2010). Thioredoxin-interacting protein links oxidative stress to inflammasome activation. Nature immunol.

[93] Gao P, He FF, Tang H, Lei CT, Chen S, Meng XF, et al. NADPH oxidase-induced NALP3 inflammasome activation is driven by thioredoxin-interacting protein which contributes to podocyte injury in hyperglycemia. J Diabetes Res. 2015;2015:504761. 10.1155/2015/504761. 10.1155/2015/504761 PMC4365330 25834832

[94] Hayashi T, Fujiwara S, Morishita Y, Kusunoki Y, Nakashima E, Nakanishi S (2003). HLA haplotype is associated with diabetes among atomic bomb survivors. Hum Immunol.

[95] Sipponen P, Maaroos HI (2015). Chronic gastritis. Scand J Gastroenterol.

[96] Yun HG, Kim HY, Kim DY, Lim YJ (2015). Successful treatment of intractable bleeding caused by radiation-induced hemorrhagic gastritis using oral prednisolone: a case report. Cancer Res Treat.

[97] Ghobadi A, Shirazi A, Najafi M, Kahkesh MH, Rezapoor S (2017). Melatonin ameliorates radiation-induced oxidative stress at targeted and nontargeted lung tissue. J Med Phys.

[98] Zhang L, Xie XY, Wang Y, Wang YH, Chen Y, Ren ZG (2012). Treatment of radiation-induced hemorrhagic gastritis with prednisolone: a case report. World J Gastroenterol.

[99] Kawata K, Kobayashi Y, Souda K, Kawamura K, Takahashi Y, Noritake H (2011). Hemorrhagic radiation gastritis successfully treated with repeated intra-arterial steroid infusions. Clinl J Gastroenterol.

[100] Graziani C, Hegde S, Mw S (2014). Radiation recall gastritis secondary to Erlotinib in a patient with pancreatic cancer. Anticancer Res.

[101] Ben-Amotz A, Yatziv S, Sela M, Greenberg S, Rachmilevich B, Shwarzman M (1998). Effect of natural beta-carotene supplementation in children exposed to radiation from the Chernobyl accident. Radiat Environ Biophys.

[102] Brown SL, Kolozsvary A, Liu J, Jenrow KA, Ryu S, Kim JH (2010). Antioxidant diet supplementation starting 24 hours after exposure reduces radiation lethality. Radiat Res.

[103] Jia D, Koonce NA, Griffin RJ, Jackson C, Corry PM (2010). Prevention and mitigation of acute death of mice after abdominal irradiation by the antioxidant N-acetyl-cysteine (NAC). Radiat Res.

[104] Bagheri H, Rezapour S, Najafi M, Motevaseli E, Shekarchi B, Cheki M, et al. Protection against radiation-induced micronuclei in rat bone marrow erythrocytes by Curcumin and selenium L-methionine. Iran J Med Sci. 2018. https://ijms.sums.ac.ir/index.php/IJMS/article/view/3771. PMC6230935 30510341

[105] Sieber F, Muir SA, Cohen EP, North PE, Fish BL, Irving AA (2009). High-dose selenium for the mitigation of radiation injury: a pilot study in a rat model. Radiat Res.

[106] Sieber F, Muir SA, Cohen EP, Fish BL, Mäder M, Schock AM (2011). Dietary selenium for the mitigation of radiation injury: effects of selenium dose escalation and timing of supplementation. Radiat Res.

[107] Son Y, Lee HJ, Rho JK, Chung SY, Lee CG, Yang K (2015). The ameliorative effect of silibinin against radiation-induced lung injury: protection of normal tissue without decreasing therapeutic efficacy in lung cancer. BMC Pulm Med.

[108] Yahyapour R, Amini P, Rezapoor S, Rezaeyan A, Farhood B, Cheki M, et al. Targeting of inflammation for radiation protection and mitigation. Curr Mol Pharmacol. 2018. 10.2174/1874467210666171108165641. PMID: 29119941. 10.2174/1874467210666171108165641 29119941

[109] Cheki M, Yahyapour R, Farhood B, Rezaeyan A, Shabeeb D, Amini P, Rezapoor S, Najafi M. COX-2 in radiotherapy; a potential target for radioprotection and radiosensitization. Curr Mol Pharmacol. 2018. 10.2174/1874467211666180219102520. PMID: 29468988 10.2174/1874467211666180219102520 29468988

